# The experience of living alone as an older woman in the UK during the Covid pandemic: an interpretative phenomenological analysis

**DOI:** 10.1186/s12889-023-14988-2

**Published:** 2023-02-04

**Authors:** Cat Forward, Hafiz T. A. Khan, Pauline Fox

**Affiliations:** 1 The College of Nursing, Midwifery and Healthcare, University of West London, London, TW8 9GA UK; 2 Present address: NIHR Health and Social Care Work Research Unit, The Policy Institute, King’s College London, Virginia Woolf Building, 22 Kingsway, Strand, London, WC2B 6NR UK; 3 Public Health Group, College of Nursing, Midwifery and Healthcare, University of West London, London, TW8 9GA UK; 4 The Graduate Centre, University of West London, London, W5 5RF UK

**Keywords:** Lived experience, Lone-dwelling, Covid

## Abstract

**Background and objectives:**

More people are living alone across the life course: in later life this can have implications for practical and psychosocial support. The Covid pandemic emphasised the importance of this when the UK government restricted movement outside of households to limit the spread of disease. This had important ramifications regarding social contact and practical support. The objectives of this study were to explore the experience of older women living alone during this time, with a focus on health and wellbeing.

**Research design and methods:**

This study used an Interpretative Phenomenological approach. Semi-structured interviews were undertaken with seven women (aged 65 +), living alone in the UK. Interviews were carried out between May and October 2020. Interpretative Phenomenological Analysis was used to analyse the transcripts.

**Results:**

Findings show that life course events shaped how living alone was experienced in later life. Convergences and divergences in lived experience were identified. Three superordinate themes emerged from the Interpretative Phenomenological Analysis: *Productivity, Ownership,* and *Interconnectedness*.

**Discussion and implications:**

Findings highlight the importance of life course events in shaping the experience of later life. They also provide a better understanding of the lived experience of living alone as an older woman, increasing knowledge of this group and how living alone can affect health and wellbeing. Implications for research and practice are discussed, such as the importance of recognising the specific support needs for this group in later life, and the need for further knowledge about groups whose needs are not met by standard practice.

**Supplementary Information:**

The online version contains supplementary material available at 10.1186/s12889-023-14988-2.

## Introduction

### The rise of living alone

A notable change in household compositions in the UK, as in many other countries, is the rise of people living alone [1]. As social and physical environments have been shown to influence later life experiences, this rise in lone dwelling is an important area of ageing studies [2, 3]. Living alone has implications for the level of practical support on hand in later life in addition to the nature of psychosocial support available. Living alone has been unhelpfully conflated with social isolation which is a distinct but related issue [4]. However, as an aspect of the social environment of ageing, living alone requires a better understanding as the health outcomes of those living alone can be poorer [5–7]. It also is a key component of the ‘Ageing-in-place’ approach adopted by many governments which aims to support people to live in their own homes in later life [8–10]. Living alone does not predict poorer health and wellbeing outcomes for all [11], and gender is one factor which has been shown to influence the experience of later life and health and wellbeing outcomes [12]. Existing qualitative research in this area tends to focus on those living with health conditions [13, 14] rather than how gender might shape this experience.

### Women and ageing

As the experience of ageing has been shown to be gendered, it is important to gain a better understanding of the nature of later life for different genders rather than treat older adults as a gender-neutral group [15, 16]. Ageing is an embodied experience both in terms of a bodily experience of the ageing process, and the way in which sociocultural norms and values differ in their treatment of men and women throughout the life course [17, 18].This indicates that research should attend to the specific and yet heterogenous experience of ageing for different genders to explore how this embodied experience can shape the quality of life in later years. Another aspect of later life which is gendered is the effects of governmental policy on the experience of older adults. Ostensibly gender-neutral policies can, in practice, be gender-blind and therefore result in financial and health inequalities in later life. One example is pension guidelines which penalise women for breaks in paid employment due to unpaid childcare [19, 20]. This means that in later life, women can be at a significant financial disadvantage, leading to socio-economic inequalities which can impact of health and wellbeing. Similarly, health research which often does not include women, or which does not distinguish between genders risks being irrelevant for women as it does not reflect important gender differences [21]. Older women experience more years of poor health and disability than men, despite longer life expectancies [22]. There is a need for research which highlights the specific nature of later life for women to reduce the current inequalities in health outcomes and experience for older women (Ko et al., 2019; Yang et al., 2020). Life course events, such as retirement, have been shown to influence the experience and health and wellbeing of older adults differently depending on their gender [23] and this has been shown to stark effect with the Covid pandemic which highlighted and exacerbated many social issues. The research presented here forms part of a larger study which explored in detail the health and wellbeing of older women living alone. This group has been shown to be more vulnerable to poorer health and wellbeing outcomes and therefore research is indicated to understand and address this [8, 9, 24].

### Covid and living alone

The Covid pandemic led to lifestyle changes globally that were reflected in the UK. The government implemented a national ‘lockdown’ restricting movement outside the household. These restrictions were in place for several months in 2020 and 2021 and are argued to have helped limit disease transmission. However, the psychosocial impact of such limitations is still being investigated. Many of the government policies around this time made assumptions about households which meant that these lockdowns posed different challenges for those who fell outside these assumptions [25]. Those who live alone are likely to have been disproportionately affected and evidence is starting to emerge regarding the effects this might have on longer-term health and wellbeing [26–29]. There is limited evidence of the experience of older women who live alone in the UK and therefore this paper will add to knowledge in this area [8]. Presented in this paper are the findings from interviews with seven women over the age of 65 who were interviewed about their experience of living alone in the UK during the Covid pandemic.

### Aim of the study

To explore the lived experience of living alone as an older woman (aged 65 +) in the UK during the Covid pandemic.

## Methodology

This study sought to better understand the lived experience of living alone, therefore an approach was chosen to best address this aim. Interpretative Phenomenological Analysis (IPA) seeks to understand the lived experience of a phenomenon, in this case that of living alone. IPA is a method which draws on the rich philosophy of phenomenology which concerns itself with the exploration of an individual’s experience. This a research approach used across health and social research disciplines to develop a rich, deep understanding of a topic [30–32].. It acknowledges the researcher as an interpreter of experience shared by participants. The approach incorporates aspects of hermeneutics by engaging with the process of interpretation [33, 34]. Typically, it uses a small, homogenous sample to gain a deeper insight, rather than a larger sample which would give more superficial data and analysis.

### Data gathering and analysis

The lead researcher advertised to potential gatekeepers, such as colleagues, participants through the social media networks Twitter and Facebook using sampling based on the predetermined inclusion criteria to ensure the research aims were met Inclusion criteria were women, over the age of 65, living alone in the UK at the time of the study, English speakers and able to give informed consent. Participants who did not meet these criteria were not included in the study. Recruitment was made more difficult as it took place after the first Covid ‘lockdown’ in the UK meaning many people were still cautious to socialise. The participants included were among a small number who responded to the advertisement and met the inclusion criteria while representing a range of ages and backgrounds within a relatively homogenous socio-cultural group. Participants either contacted the first author directly or were put in touch via others who had seen the advertisements. More detailed information was provided about the study and the interview process to those who contacted the first author with interest in taking part.

Seven participants agreed to be interviewed individually. Research using an IPA approach commonly has small numbers of participants due to the rich nature of the data sought [35]. Audio-recorded, semi-structured interviews were carried out face-to-face (*n* = 6) or using video conferencing software (*n* = 1), between July and October 2020 and took an hour on average. The topic guide is included as additional material. The interview questions were designed to be open ended and approaching the topic of living alone from a ‘sideways’ angle [32] to encourage an exploration of the topic in a way meaningful to the participant. Recordings were transcribed and analysed using Interpretative Phenomenological Analysis by the first author.Transcripts were analysed manually as is usual for studies with an ideographic focus [36]. Interview data were analysed for experiential aspects and important themes in an iterative process, refining and grouping the themes identified at each stage. Transcripts were read through repeatedly, looking for references to aspects of living alone during this time. With each reading, key phrases or words were picked out and then grouped and paraphrased to start to develop them into broader themes. Initially, the themes were developed within each individual case before being considered across the sample. This means that analysis moves between the individual and the general to develop themes which can be found across the cases while acknowledge the importance of the individual experience. Table 2 shows the development of the superordinate themes and subthemes developed. For quality purposes, an audit trail was created and checked by the other authors and a selection of transcripts (*n* = 3) were checked for consistency of analysis by the second author. Finally, a reflective diary was kept by the first author to address issues around bias and bracketing as is common in IPA research and shared with the second author [32, 37] As IPA acknowledges the hermeneutics involved by the researcher engaging in the communication of experiences by the participants, it is important to reflect on the position of the researcher to the topic. At the time of data gathering I was a 36-year-old woman, cohabiting with a male partner. I was trained and had worked as an Occupational Therapist. I had not experienced living alone or of being an older woman but had the potential to do so. I was also of a different generation to those I interviewed but a similar socio-economic background. My clinical practice had meant that I had often met older women who lived alone, primarily in South London, and of many various backgrounds and circumstances. This meant that I was aware of a multitude of experiences which could exist as an older woman living alone and perhaps had an openness to this. It also meant that I had to consciously ‘block’ my therapeutic approach and shift to that of researcher.

#### Ethical considerations

The study was given ethical approval from the host institution. Informed consent was obtained from all participants and any identifying information was kept secure. The names used are pseudonyms.

## Results

Table 1 summarises the participants’ demographic information, from data gathered during the interviews. The sample is relatively homogenous in terms of ethnicity and age: all being White British and ‘young-old’ [38]. Education level varied but all were retired from their primary, full-time paid employment of a white-collar type. Most were involved with voluntary work, which was limited by the pandemic, but five remained involved to some degree. The only one of the participants who did not own her own home resided in a property owned by the religious order to which she belonged. This appeared a secure arrangement which seemed comparable to own-ownership in terms of stability but perhaps alleviated some of the level of responsibility expressed by the other participants in their management of the home.

**Table 1:** Description of participants

*Name (pseudonym)*	*Age*	*Marital status*	*Housing tenure*	*Level of education*	*Ethnicity*	*Volunteering status*	*Time living alone*
Denise	65	Single	Owner occupied	PG Dip	White British	Volunteering	28 years
Cathy	80	Single	Rented privately or with a job or business	MSc	White British	Volunteering	20 years
Wendy	73	Widowed	Owner occupied	School level	White British	Volunteering	4 months
Angela	74	Widowed	Owner occupied	School level	White British	Not currently volunteering	10 years
Diane	74	Separated	Owner occupied	BA	White British	Volunteering	< 1 year
Kim	74	Surviving partner	Owner occupied	MSc	White British	Volunteering	7 years
Janet	66	Widowed	Owner occupied	School level	White British	Has not volunteered	< 1 year

*PG Dip* Post-graduate diploma, *MSc* Master of science, *BA* Bachelor’s degree

The reasons and level of choice around living alone varied. The most common reason for living alone was bereavement of life partner or husband, one of the women lived alone following the breakdown of a relationship and another had lived alone having never been partnered. Only one of the participants (Cathy) made an active choice to live alone. This likely influenced the experience of living alone which is reflected in the themes discussed below.

### Productivity

As can be seen in Table 2, the theme of productivity encompasses several ideas relating to time use, including both the presence and absence of such activities. In this context the theme of productivity relates to an activity which may be considered productive, as opposed to leisure or self-care. Volunteering, paid work, housework or unpaid care work may all fall under this category. For all seven of the women this was present to some extent providing a convergence of experience; divergences were notable in how this sense of productivity manifested. There were also divergences in the ways in which the element of productivity related to their experience of living alone and in turn, to their health and wellbeing. The women were retired from their primary paid employment and engaged to varying degrees in alternative means of productivity.

**Table 2:** Summary of final themes

Superordinate theme	Subthemes	Support from data
Productivity	Role / structure / purpose / routine / absence	“…made me think what, what purpose have I got? I don’t work, I don’t…but volunteering is so important…” (Denise) “… so I don’t do much unless I go to a class so I’m a member of various groups and that’s what I would normally do but as they’re not going at the moment I’m not doing anything…” (Diane)
Ownership	Choice / health / decision-making / practicalities	“I’ve got to do something, but I don’t know what I am going to do… But there again it’s up to me, in the end. No-one can tell me what to do.” (Janet) “… of course, I’m wondering what will happen cos … would I rather stay here [current home] and be peaceful and have people in y’know like I do now really you know just as friends but not being with people in constantly if you see what I mean?” (Kim)
Interconnectedness	Continuity / ruptures / adaptation / reciprocity	“And I lost all the volunteering job cos they all shut obviously and all this meeting friends, my whole life in fact I lost.” (Diane) “But as I say I’ve got through it a bit and you just have to make a different path for yourself I suppose.” (Angela)

The types of productivity the women discussed included unstructured household tasks such as cleaning or gardening in addition to more formal arrangements such as volunteering, or community work. Unpaid work, such as Angela’s care work for a friend’s son, provided a similar role to formal arrangements such as volunteering, in terms of role and structure. Several women acknowledged the advantages of formal volunteering themselves, stating that ‘*you get the benefit*,’ (Wendy). Angela went so far as to describe the choice to volunteer at her local hospital as ‘*selfish*,’ as she understood herself to be benefitting from the activity, perhaps wanting to downplay any ideas of ‘*do-gooding’*.

The role of activity in general was also interesting in terms of providing a structure to days and weeks when paid employment no longer did this. This included classes, meeting friends, attending galleries, or engaging in community work. Cathy and Denise both spoke of having routines which gave shape to their lives but also contributed to their sense of self and identity. Denise described this as ‘*purpose,’* suggesting the importance of feeling involved in activities which are contributing to the local community. The development of new routines, especially following the loss of a partner, was something which was important for several of the women as part of the process of coming to terms with a loss. This was highlighted by the experience of Janet who was yet to establish this for herself and consequently found herself in a sort of limbo following the loss of her partner and decision to retire.

This sense of life being ‘on-hold’ was present for all women in some form due to the pandemic. This disease is the first major disruption in the UK for many years, certainly within the participants’ lifetimes. The lockdowns enforced by the UK government, as with many governments worldwide, to limit the spread of the disease meant that most people were expected to remain at home except for essential tasks. This meant that the volunteering and the other community activities were on hold for these women and so too their structure and purpose. The indication from the interviews was that this loss of structure posed a threat in terms of identity and wellbeing. Kim described the lockdown period as ‘*grim,*’ as she discussed her attempts to keep busy around the house and maintain social contact through telephone calls and text messaging. Angela and Diane had significant health problems and were advised to remain isolated to reduce their risk but found the impact on their mental wellbeing such that they negotiated a way of going out for walks during quiet times of the day. While the Covid pandemic is a particularly extreme example of a disruption to routine and productivity, it does highlight the value of engaging in activities, especially activities out of the home, to contribute to health and wellbeing.

### Ownership

The theme of ownership encompasses several aspects of ideas around taking control or making decisions and reflects both positive and negative facets of this. The idea of choice refers to the desire and ability to make choices around time-use, the people someone spends time with, where to live or even the decision to live alone. This was something which came up in all the interviews and was central to several of the experiences shared. There was a value placed on the ability to make their own choices although the other aspect of this was the uncertainty at times that the ‘right’ choice was being made.

Cathy’s decision to seek out the opportunity to live alone is central to her experience and enjoyment of this phenomenon. Denise and Diane both express a strong desire to exert choice over their time-use and this increases the value of the activities they undertake. Another side to this is that there are situations in which choices are limited or not available. This could include the reasons for living alone which was not an active choice for any of the women except for Cathy. A limitation on options of time-use such as those imposed by the national lockdowns relating to the Covid pandemic was also another example of a restriction of choice. Although these are extreme examples, they illustrate how a loss of control or choice over a situation had a negative effect on wellbeing.

The more negative aspect of this idea around choice and ownership is the sense of indecision it presents when a person has options to choose from or decisions to make but finds it difficult to pick the ‘right’ one. This was often coupled with a situation in which these choices were not perhaps the preferred ones, such as the options around downsizing for Janet and Kim which were essentially related to their bereavements. Aside from the connection with a bereavement itself, it also seemed to be that much of the difficulty around making a decision, was related to their loss of a partner-as-confidante with whom decision-making was often shared. This seems to indicate a stressor for those living alone which may not be experienced in the same way by those cohabiting. Both had robust friendship groups or support networks outside of their household but there was a sense that their partners had been more depended on for advice or decision making.

This idea of ownership also related quite specifically to difficulties and stresses experienced as a result of managing and running a home alone. All the women shared experiences of challenges posed by the upkeep of the home and garden. The women were all in secure and appropriate housing and appeared to be financially secure in terms of being able to afford essential maintenance. All owned their own homes except Cathy whose property was owned by her employer, and she appeared to experience the least concerns around managing a home, perhaps due to the support the employer would provide with this as a way of sharing responsibility. For the other six women, issues around housing maintenance or DIY tasks seemed to present a stressor either in executing the tasks themselves or managing to find someone trustworthy to come into their home. Difficulty taking ownership with some of the practicalities of running a home was common to many of the experiences shared in these interviews and illustrates a challenge to a sense of self-efficacy. Throughout the interviews, these situations were stressors to women who had been successful in other aspects of their life such as employment and childrearing. As such these experiences appeared to negatively affect their wellbeing, however, it does indicate a way in which suitable support services such as voluntary organisations could improve the experience of living alone, through enabling a sense of self-efficacy.

Finally, several of the women presented experiences in which the sense of ownership was related to an active engagement in health promotion or maintenance. By taking control of aspects of their lifestyle which affect their health and wellbeing, they sought to further protect their health and independence in later life. Cathy spoke in detail of watching what she ate and taking regular exercise in order to ensure bowel health and more general wellbeing. Angela and Diane, who experienced Rheumatoid Arthritis and Systemic Scleroderma respectively, both acknowledged an awareness of needing to manage their conditions through methods such as medication adherence or modifying activities. Others who reported good health, such as Kim and Denise, actively sought to maintain this through exercise and diet, with Denise hoping to avoid the need for any ‘*tablets*.’ This is contrasted with the experience of Janet who seems to be less concerned about this aspect of her lifestyle but acknowledged it as an area of her life in which she should make changes. For all of the women, there was an acknowledgement of the role in which health behaviours could contribute to health and wellbeing indicating a certain level of self-efficacy, reinforcing these ideas of choice and control.

### Interconnectedness

The final theme of interconnectedness presents both as a presence and an absence, much like the other two themes. It refers to the social relationships the women had with family, friends, and the broader community. All seven of the women seemed to have robust social networks, particularly in terms of friendship groups. Family connections were important to several of the women but not all. The women all had social connections which they maintained through time and across geographical boundaries. The recent Covid restrictions provided a challenge in this respect but the use of internet-based technology, such as Zoom, FaceTime and email, helped to provide a sense of continuity in connectivity and support. This appeared to benefit the wellbeing of the women as they described experiences whereby the use of technology had enabled them to promote wellbeing through social connection. They did, however, report some misgivings and frustrations with aspects of technology: Denise describes missing seeing people in real life, Diane describes her frustration with features of Zoom as does Kim and several women expressed preference for telephone calls with which they were more familiar. It seemed clear that there were certainly benefits to the use of internet and related technologies particularly during such an extreme period of social isolation. However, the response to and use of technology appeared to vary depending on the purpose, frequency and whether it was attempting to replace or augment more traditional social contact.

Covid was not the only event which caused ruptures to social contact. Most obviously, bereavements, especially the loss of a partner, were also significant and influenced how the women experienced living alone. Both Denise and Wendy experienced bereavements (of their mother and husband respectively) during the first Covid lockdown which compounded the sense of rupture. Rules at this time in the UK limited the size of gatherings and this affected their ability to carry out the planned funeral arrangements they had expected. This experience seemed detrimental to wellbeing on several levels. While they accepted it as necessary to stop the spread of the disease, they expressed a sense that their ability to grieve was affected and that the process of carrying out a funeral with so little presence of friends and family made the experience all the more distressing. Other interruptions included the ending of relationships such as Diane’s experience of a relationship ending after 40 years. This was, in many ways, a loss which echoed those of widowhood but was complicated both by emotions such as rejection and by the fact she was uncertain whether to remain in touch with her ex-partner. This required her to engage in a period of adaptation, in which she was still engaged, as she tried to renegotiate the relationship and whether it was beneficial to her or not.

The sense of adaptation in terms of maintaining connectivity was consistent throughout. In addition to the uptake of technology use during Covid, changing events over life courses meant that a level of flexibility was required to maintain friendships and other social contact. Several women had friendships spread across the UK and the globe which they maintain through emails, phone calls and visits. Wendy described friendships which she had kept up since her schooldays including one man for whom she was now Power of Attorney. This reflects the ways in which relationships can change and adapt over time out of necessity as well as choice. Denise experienced changes in friendships over the years, notably if friends became newly single or married which affected the dynamics of their relationship as the needs of her friends changed in terms of support or availability. Throughout the interviews there was a sense of openness to dealing with these changes which was associated with maintaining valuable support networks. Another example of adaptation is the use of volunteering or organised social groups such as the University of the Third Age to provide social connection, especially in retirement. Denise and Diane described a deliberate joining of such activities to promote their mental wellbeing.

All the women expressed a need to engage in activities outside of the home, suggesting their wellbeing was the better for it and there was a sense that while the women were supported by good social networks, they valued the sense of reciprocity gained by providing care or support to others. In addition to formal volunteer work, Angela had also been involved in providing unpaid care for a friend’s son as well as regular time spent with her grandchildren. There was a feeling from this, and from the other women who provided support to others, that there was a value in the sense of a broader reciprocity: that they could receive support when needed but that they were also in a position to provide support to others. This sense of reciprocity appeared to be linked with the ideas of role and purpose attached to productivity and underlined the importance of this is terms of supporting a sense of self. During the period of the lockdown, several of the women were required to remain at home due to health reasons and accept help from others. Their sense of self appeared challenged by this dynamic, perhaps as they were unable to fulfil a perceived social expectation for reciprocity.

## Discussion

The analysis of the interview data found three superordinate themes of Productivity, Ownership, and Interconnectedness. This data and the resulting themes give an insight into the experience of living alone as an older woman during the Covid pandemic. These themes echo issues found in the wider literature and are discussed here within that context.

### Productivity

The value placed on being productive, such as that seen by Denise and Cathy, is consistent with existing literature which highlights the value of continued engagement in later life in an occupation of sorts whether volunteering or lifelong learning [39–41]. The benefits of participating in such activities are well documented, even when outcomes are controlled for initial health status [42]. These benefits include increased social contact, sense of purpose and a routine or structure. These are often features of paid employment which people lack in retirement. This was acknowledged by Angela when she described her volunteering at the hospital as ‘*selfish*,’ as she took up the work for the psychosocial benefits to herself.

This need to be involved in productive tasks might also reflect a value judgement in terms of the types of work a person does, and the value attached to it, something which can be addressed within the context of a feminist gerontology. This approach critiques the idea of a person’s value being established via their role as a productive member of society especially in terms of a member of the labour market within a capitalist culture [43]. This is particularly relevant for women who are often engaged in care work or household work which is devalued under this system [44, 45].It becomes relevant in later life during retirement as a person’s role and sense of self are challenged by removal from the labour market. This is indicated by Denise and Janet in these interviews, confirming that which is already demonstrated in the literature in terms of the value of an ‘*occupation*’ [46, 47].

The need to social distance due to the Covid pandemic meant that formal volunteering activities or other informal support activities were lost to many people such as Denise and Wendy. The early evidence indicates that this loss of role and social interaction had significant impacts on mental wellbeing for the volunteers as well as the recipients of such services [48]. One element of the theme of productivity was that of routine and structure which is provided by having formal or regular activities in place. In addition to Covid, another challenge to this structure was through bereavement during which the women described having to restructure their time as part of the process of adaptation which is consistent with previous research [49].

### Ownership

The theme of ownership related to ideas around control and choice. This is commensurate with much of the literature in that a sense of choice over a person’s own situation or aspects of an individual’s life was related to increased wellbeing [50–53]. This was evident to some degree in the experiences shared in this study, as the women valued the freedom to make their own choices and appeared to find it challenging when choices were limited or denied such as in the case of Covid restrictions or ill health.

The challenge to this sense of ownership was that of decision-making without a partner to guide the process. This could be for larger decisions such as where to live but also smaller choices such as those around home maintenance. This came up for all of the women except Cathy who did not own her own home. The relationship between environment and wellbeing is well documented [54, 55], but it is interesting that the challenge of managing a home can become a stressor in addition to a home being a resource or a positive entity. This is something which has been seen in some studies, but which is underexplored in the literature [56].

The way in which the women interviewed discussed their ownership around health management reflected several ideas evident in the literature around health or active ageing [57]. Ideas around eating well or maintaining physical fitness suggest that the approaches to healthy ageing put forward in evidence and policy are evident at some level in this group. This can be seen in Cathy and Angela’s awareness of diet and exercise in managing existing health conditions. However, as white middle-class women, the participants are perhaps in a subgroup of the population who are in situations which enable them to make such choices. This tension between an individual’s ownership over health and the influence of political, social, and cultural context is one which may be less evident in such a sample, but which needs addressing in future research [58]. Exploration of other groups of older women would be particularly useful in this regard to explore the cultural and contextual variations likely to exist as lifelong inequalities challenge this active ageing approach [59, 60].

### Interconnectedness

The theme of interconnectedness encompassed ideas around personal and wider social connections. Research often focuses on familial support in later life, but evidence is growing that friendships are often of equal if not greater importance and this is something especially relevant for women who live alone [61, 62]. The experiences shared in these interviews suggest that friendships were important to all the women, but especially to those who did not have children or close family support. The importance of social support was highlighted during the Covid-19 pandemic and the lockdowns put in place. These women experienced this extraordinary time in history at a certain age and time in their lives which may impact how they dealt with the restrictions. One suggestion could be that older adults, having experienced adversity over a life course, may be better equipped to deal with another period of difficulty. Early evidence shows generational variances in the impact of the social isolation and coping abilities, but further research is needed to fully understand these [63, 64]. Increased use of technology to augment social connections during the pandemic has been evidenced in emerging literature but the effect of this shift has yet to be seen and the indications are that it cannot replace in-person contact [65].This mixed approach to technology and the resulting sense of connection reflects the results of existing research which exist regarding the use of the internet by older adults [66–68].

The engagement in volunteering or other structured activities appeared valued by Denise, Wendy, and Angela for the sense of social connectedness in addition to contributing to the local community. Existing literature suggests that women are more likely than men to engage in and benefit from social activities, particularly those out of the home, in terms of their health and wellbeing [69, 70]. Volunteering as a route to social connectedness is well-recognised. This is consistent with evidence which suggests that there is a shift towards formal social activities in later life, perhaps to compensate for reduced informal activities: although for many of the participants it appeared complementary rather than compensatory [71].

The sense of reciprocity within social networks, which was seen in the experiences shared in this study, by Angela and Cathy for example, has been shown in the literature to improve wellbeing when compared to those just receiving support and has led to an increase in interest in encouraging volunteering as a health promotion or public health issue [72]There is certainly a value in this work for providing a role or structure and in promoting social connectedness [73], but there are concerns, particularly in critical thinking, that care work generally falls most often to women and is undervalued with a risk of detrimental effects to wellbeing [74]. This suggests that for older women particularly, more research is required into volunteering or more informal unpaid work as determinants of health and wellbeing.

## Conclusion

This paper presents findings from the Interpretative Phenomenological Analysis of seven interviews carried out to increase knowledge of the experience of living alone as an older woman in the UK.

The research presents valuable and novel findings regarding women’s experience of living alone during the COVID pandemic at a unique time in UK history. Prior to this, little was known about this specific experience. Carried out during the Covid pandemic in 2020, the interviews provide an insight into the phenomenon of living alone as an older woman at a unique time in UK history. Despite the unusual circumstances, the experiences reflected many of the themes which arise in the existing literature, giving credence to the results and the commonality of human experience. The study gives an insight into how the themes and experiences known thus far are challenged or maintained during a period of uncertainty such as a pandemic.

From the individual experiences, convergences and divergences have been identified and three superordinate themes emerged from the data: *Productivity, Ownership,* and *Interconnectedness*. These themes have been discussed within the context of the data and existing literature, suggesting areas for future research relating not only to demographic characteristics but also to volunteering and unpaid care work, as the relationship between ‘work’ in this context and health and wellbeing appears a complex one, especially considering debates around the politics of care and the political economy of ageing [75, 76].

### Strengths and limitations

This paper is based on findings from an IPA study exploring the lived experience of older women living alone in the UK during the Covid-19 pandemic. It presents valuable findings regarding this experience which were previously unexplored. The homogeneity of the group can be considered a limitation; however, it is a key part of the IPA approach. It highlights the need for further research of other groups of older people living alone such as older men, those who are gender non-conforming or trans and women of other socio-economic or cultural backgrounds. The strength of this research is its focus on the lived experience of older women which counterbalances more gender-neutral or gender-blind approaches [77].

## Supplementary Information

**Additional file 1:** **Supplemental material.** Interview Schedule.

## Data Availability

The anonymised data from these interviews are not publicly available due to the nature of the consent gained by participants, however, they are held by the corresponding author and can be made available on reasonable request.
