# Supplementary material for: The experience of living alone as an older woman in the UK during the Covid pandemic: an interpretative phenomenological analysis
Source: BMC Public Health. 2023 Feb 4;23:244. doi: 10.1186/s12889-023-14988-2 (PMC9898705; doi:10.1186/s12889-023-14988-2)
Supplement: Supplementary file 1 — Additional file 1: Supplemental material. Interview Schedule. [file 12889_2023_14988_MOESM1_ESM.docx]

**Supplemental material: Interview Schedule**

Can you tell me a bit about how you came to live here?

Have you made many changes to the property since you have been here?

What would you change about the house/flat if you could?

Have you ever thought of living anywhere else?

Can you tell me about any visitors or help you might have around the house?

Could you talk a bit about your routine? Daily, weekly?

Can you talk to me a bit about your health generally? You do not need to reveal anything you do not wish to.

Can you tell me much about the local area and your connection with it?

Could you talk a little about the things which you value about living alone?
